# The unfolded protein response and its potential role in Huntington's disease elucidated by a systems biology approach

**DOI:** 10.12688/f1000research.6358.2

**Published:** 2016-03-02

**Authors:** Ravi Kiran Reddy Kalathur, Joaquin Giner-Lamia, Susana Machado, Tania Barata, Kameshwar R S Ayasolla, Matthias E. Futschik

**Affiliations:** 1 Centre for Biomedical Research, University of Algarve, Faro, 8005-139, Portugal; 2 Centre of Marine Sciences, University of Algarve, Faro, 8005-139, Portugal

**Keywords:** Unfolded protein response (UPR), Huntington's disease, Apoptosis, UPR interactome, HTT interactome

## Abstract

Huntington ´s disease (HD) is a progressive, neurodegenerative disease with a fatal outcome. Although the disease-causing gene (huntingtin) has been known for over 20 years, the exact mechanisms leading to neuronal cell death are still controversial. One potential mechanism contributing to the massive loss of neurons observed in the brain of HD patients could be the unfolded protein response (UPR) activated by accumulation of misfolded proteins in the endoplasmic reticulum (ER). As an adaptive response to counter-balance accumulation of un- or misfolded proteins, the UPR upregulates transcription of chaperones, temporarily attenuates new translation, and activates protein degradation via the proteasome. However, persistent ER stress and an activated UPR can also cause apoptotic cell death. Although different studies have indicated a role for the UPR in HD, the evidence remains inconclusive. Here, we present extensive bioinformatic analyses that revealed UPR activation in different experimental HD models based on transcriptomic data. Accordingly, we have identified 53 genes, including RAB5A, HMGB1, CTNNB1, DNM1, TUBB, TSG101, EEF2, DYNC1H1, SLC12A5, ATG5, AKT1, CASP7 and SYVN1 that provide a potential link between UPR and HD. To further elucidate the potential role of UPR as a disease-relevant process, we examined its connection to apoptosis based on molecular interaction data, and identified a set of 40 genes including ADD1, HSP90B1, IKBKB, IKBKG, RPS3A and LMNB1, which seem to be at the crossroads between these two important cellular processes. Remarkably, we also found strong correlation of UPR gene expression with the length of the polyglutamine tract of Huntingtin, which is a critical determinant of age of disease onset in human HD patients pointing to the UPR as a promising target for therapeutic intervention. The study is complemented by a newly developed web-portal called UPR-HD (http://uprhd.sysbiolab.eu) that enables visualization and interactive analysis of UPR-associated gene expression across various HD models.

## Introduction

Huntington’s disease (HD) is an autosomal-dominant neurodegenerative disorder. Its symptoms include loss of motor control, cognitive decline, and behavioural abnormalities. In most cases, the onset of the disease occurs between the age of 35 and 50. The outcome is always fatal with a life expectancy following the disease onset of around 20 years.

The treatment of HD has remained symptomatic, as currently there is still no cure. The cause of HD is a mutation in a single gene called huntingtin ( *HTT*). In HD patients, an expansion of the CAG repeat in exon 1 of huntingtin has been identified ^1^. This mutation results in an extended stretch of polyglutamine close to the N-terminus of the Huntingtin protein (HTT), which is involved in multiple molecular functions ^2, 3^. Although the molecular cause has been known now for over 20 years, the exact mechanisms leading to the observed massive cell death of neurons in the caudate nucleus of HD patients still await full clarification. A variety of processes such as excitotoxicity ^4, 5^, protein aggregation ^6– 9^ and transcriptional dysregulation ^10^ have been suggested to contribute to neurodegeneration in HD. More recently, several studies have indicated that the unfolded protein response (UPR) might be implicated in neurodegenerative diseases including HD ^11– 13^.

### ER stress and UPR

**Figure 1.**
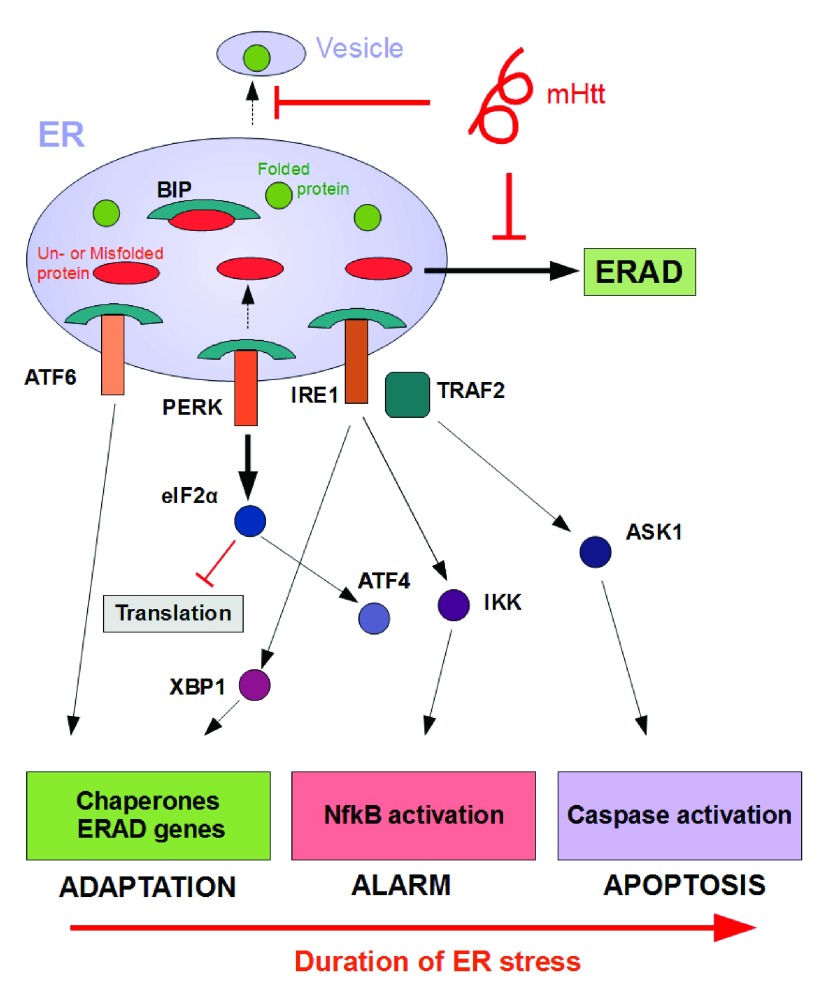
Phases of UPR activation and putative triggering of ER stress by mHTT. One explanation for how UPR sensors IRE1, PERK and ATF6 are activated is through detachment of chaperone BiP in the presence of excess un- or misfolded protein. This leads subsequently to the execution of a series of molecular processes with different effector functions ^17^. As an adaptive response, the UPR up-regulates transcription of chaperones, temporarily attenuates new translation, and activates protein degradation via the proteasome. Persistent levels of ER stress, however, may trigger inflammatory pathways as an alarm signal in addition to caspase activation, leading ultimately to the induction of apoptosis. Mutant Huntingtin (mHTT) might provoke ER stress through interference with different processes such as vesicular transport or ER-associated degradation (ERAD) resulting in accumulation of (misfolded) protein in the ER.

The endoplasmic reticulum (ER) is a crucial organelle for the correct folding and modification of numerous proteins. Upon the accumulation of unfolded or misfolded protein in the ER, several transcriptional and translational mechanisms are triggered to ensure fidelity of protein folding ^14, 15^. This stress response is better known as the UPR. In particular, the UPR stress sensors inositol-requiring enyzme1 (IRE1), activating transcription factor 6 (ATF-6) and PKR-like ER kinase (PERK) are activated in mammalian cells when the ER exceeds its capacity for correct folding. As an adaptive response to counter-balance accumulation of un- or misfolded proteins, the UPR (i) upregulates transcription of chaperones, (ii) temporarily attenuates new translation, and (iii) activates protein degradation via the proteasome ^16^. The main function of the UPR is re-establishing homeostasis by increasing the overall folding capacity. Although the primary role of UPR is an adaptive one, persistent ER stress can mediate toxicity and eventually lead to apoptosis through activation of JNK, ASK1 and caspase-12. Figure 1 depicts the different mechanisms and outcomes of UPR activation.

### UPR and HD

ER stress and UPR have been indicated for a variety of neurodegenerative disorders, where protein misfolding plays a significant role ^18– 20^. For HD, finding a direct connection appears to be an enigma at first glance, since HTT is commonly thought to be located in the cystosol or eventually in the nucleus ^21^, but not within the ER lumen. Thus, its misfolding should not trigger UPR. However, several proposals have been put forward to describe how mutant HTT (mHTT) can induce an ER stress response ^12^. For instance, experimental evidence from HD cell models suggests that cytosolic mHTT fragments strongly impair ER-associated protein degradation (ERAD), since mHTT entraps ERAD proteins ^22^. This impairs the proper protein catabolism, causing a potential accumulation of misfolded proteins in the ER, effectively interfering with its correct functioning. Another alternative route towards ER stress in HD could be the perturbation of vesicular trafficking by mHTT resulting in a general protein overload in the ER or the disturbance of ER calcium homeostasis leading to a decreased folding capacity ^12^. Moreover, HTT may even be an integral part of the ER stress response, as a reversible association of HTT via its highly conserved N-terminal domain with the ER membrane was observed ^23^. Under stress conditions, HTT is released and translocates to the nucleus, where it might impact on gene expression. Nuclear export and the subsequent re-attachment of HTT to the ER then terminate this stress response. It was suggested that such a potential control mechanism is disturbed through the nuclear accumulation of mHTT resulting in a perturbed ER in HD ^24^.

Although various lines of investigations have shown a potential role for UPR in the pathogenesis of HD, it remains difficult to assess its overall influence, given that the animal models and cell lines used in each individual study display great variability and distinct characteristics. Furthermore, most studies addressing the connection between UPR and HD focus on a small set of genes and proteins ^25, 26^. As the UPR presents a potentially important process in HD progression and a novel therapeutic target, we aimed to complement these previous studies with systematic and comprehensive bioinformatic analyses. Accordingly, using a systems biology approach, we gathered all available data and focused on detecting the activation of UPR during HD, and also on elucidating the potential connection between UPR and apoptosis in HD.

First, we assembled different sets of genes associated with the UPR and examined whether the included genes show differential expression in HD models or patients, when compared to controls. Next, we examined the promoter regions of upregulated UPR genes and detected significant enrichment of characteristic stress response elements. Additionally, we performed functional enrichment analysis on differentially expressed genes and found major biological processes implicated in UPR to be significantly over-represented. Furthermore, we assembled the UPR interactome and identified common proteins involved with apoptotic processes and interacting with HTT, since those could provide crucial links between apoptosis and HD.

## Materials and methods

### Derivation of gene sets for UPR, apoptosis and HD

Since the UPR is a complex process, it is challenging to define a unique set of associated genes. Accordingly, we compiled three alternative gene sets that are either directly or indirectly involved in UPR, gathered from distinct sources. The first termed, UPR-KEGG-GO ( *n=265*), was derived from Gene Ontology ^27^ ( http://geneontology.org/page/go-database) (RRID:nif-0000-20935) as well as Reactome ( http://www.reactome.org/) (RRID:nif-0000-03390) ^28^ and KEGG Pathway database ^29^ ( http://www.genome.jp/kegg/pathway.html) (RRID:nlx_31015) databases indicated in Table 1. The second, referred to as UPR-Interactions ( *n=281*), was generated by assembling molecular interactions of UPR components *ATF6, ATF4, DDIT3, EIFAK3, ERNI* and *XBP1* using UniHI ^30^ ( http://www.unihi.org/) (RRID:nif-0000-03609) and HDNetDB databases ( http://hdnetdb.sysbiolab.eu). The third gene set, labelled UPR-Literature ( *n=2048*) was compiled from published experimental studies ^31– 34^ performed in yeast and human cells using high-throughput techniques such as yeast two-hybrid, microarrays and ribosome profiling coupled with next generation sequencing, as well as from text-mining of the GeneCards database ^35^ ( http://www.genecards.org) (RRID:nif-0000-02879) ( Table 1).

**Table 1.** Data sources used to compile UPR gene sets.

Gene set	Sub-classification			Number of genes	Total number of unique genes
**UPR-KEGG-GO**	**Pathway**				265
	KEGG:04141: Protein processing in ER			165	
	REACTOME: Unfolded protein response			79	
	**Gene ontology**				
	GO:003043: ER-associated ubiquitin-dependent protein catabolic process			38	
	GO:0030968: endoplasmic reticulum unfolded protein response			89	
	GO:0034976: response to endoplasmic reticulum stress			142	
**UPR-Interactions**	**Interactions**				281
	ATF4			92	
	ATF6			34	
	DDIT3			73	
	EIF2AK3			13	
	ERN1			37	
	XBP1			111	
**UPR-Literature**	**Publications (High through-put experiments)**			1675	2048
	Study	Species	Method		
	Labunskyy VM *et al.* ^31^	Yeast	Ribosome profiling coupled with NGS	189	
	Travers KJ *et al.* ^32^	Yeast	DNA microarray	745	
	Jonikas MC *et al.* ^33^	Yeast	Synthetic genetic array methodology & High-throughput flow cytometry	1262	
	Christianson JC *et al.* ^34^	Human	Affinity purification, LC-MS/MS, High-throughput Y2H	75	
	**GeneCards**				
	GeneCards: ER-Stress			275	
	GeneCards: Unfolded Protein Response			325	

In order to examine the genes involved in the cross-talk between UPR and apoptosis, we derived a list of genes that are either directly or indirectly involved in apoptosis from several different sources, namely the Gene Ontology database (GO:0006915; Apoptotic process; n=431), KEGG pathway (hsa04210; Apoptosis; n=88), Reactome pathway database (REACT_578.8; Apoptosis; n=148; http://www.reactome.org/) (RRID:nif-0000-03390) and literature reviews (n=85) ^36– 40^. All genes included were annotated to be involved in the induction of apoptosis, anti-apoptosis, regulation of apoptosis or were caspases (including both activators and inhibitors).

For establishing putative links to HD, we additionally put together two other gene sets:

(i) HD Therapeutic Targets (HDTT) comprising 1033 genes. This set includes genes which were annotated by the curators of the HD Research CrossRoads database as being associated with HD based on experimental evidence, making them potential therapeutic targets. A detailed description of this gene set is provided elsewhere by Kalathur *et al.* ^41^. The list of HDTT can be accessed at http://hdtt.sysbiolab.eu/.
(ii) HTT interactors ( *HTT-int*) including 1015 genes whose proteins have been shown to interact, or to be physically associated with HTT based on a diverse range of experiments. The large number of interactors can be explained through the inclusion of high-throughput affinity purification experiments, which frequently results in the addition of indirect interactions (e.g. within complexes). This set of interactors was obtained from the HDNetDB database ( http://hdnetdb.sysbiolab.eu) and can be found in Supplementary data file 8. The annotation available in HDNetDB was used to distinguish direct and indirect interactions. In total, 234 proteins were registered as direct interactors.

### Collection and processing of HD gene expression data

All HD gene expression data used for this study were downloaded from the Gene Expression Omnibus (GEO) database ^42^ ( http://www.ncbi.nlm.nih.gov/geo/) (RRID:nif-0000-00142). These data include gene expression from human brain and blood samples, human iPSCs, mouse, rat and yeast HD models, as well as murine cell lines ( Table 2). All expression data sets were pre-processed using RMA (Robust Multi-array Average) implemented in R (available at http://www.r-project.org/) (RRID:nif-0000-10474) and analysed using several Bioconductor packages ^43, 44^ (RRID:nif-0000-10445). To enable the comparison across organisms, we mapped genes from mouse, rat, worm and yeast to orthologous human genes using HGNC Comparison of Orthology Predictions (HCOP) search tool (available online at http://www.genenames.org/cgi-bin/hcop), which is based on integrated data from HUGO Gene Nomenclature Committee (HGNC) ^45^ (RRID:nif-0000-02955).

**Table 2.** List of HD gene expression datasets used for the gene set enrichment analysis.

GEO ID	Sample	Organism	Pubmed id
GSE3790	HD (CN) vs Control	Human	16467349
GSE24250	HD vs Control (blood)	Human	21969577
GSE37547	HD-iPSC vs corrected HD iPSC	Human	22748967
GSE3621	R6/1-18w, 22w, 27w vs WT	Mouse	17696994
GSE9803	R6/2-12w vs WT	Mouse	17519223
GSE10202	CHL2-22m vs WT	Mouse	17519223
GSE9330	Ctip2-KO vs WT	Mouse	18199763
GSE18551	YAC128-12, 24m vs WT	Mouse	20089533
GSE3583	HdhQ111 vs WT	Mouse	17708681
GSE9760	mESC (CAG150)-d4, d6 vs WT	Mouse	NA
GSE12481	Neuronal-culture 82Q vs CT	Rat	18815258
GSE18644	Htt103Q vs Htt25Q	Yeast	21044956

Additionally, we evaluated an unpublished expression dataset (available at http://chdifoundation.org/datasets) generated by the CHDI Foundation. It comprises RNA-Seq measurements of striatum, cortex and liver tissue taken from 6 month old heterozygote mice with CAG lengths of 20, 80, 92, 111, 140, and 175. The availability of data for distinct repeat lengths enables the examination whether expression changes are dependent on the length of the HTT polyglutamine tract. Spearman correlation between CAG number and gene expression measured as fragments per kilobase of exon per million reads mapped (FPKM) was calculated for each gene. False discovery rates (fdr) for positive and negative correlation were estimated by comparing the observed correlation coefficients with the distribution of correlation coefficients obtained for permutated data. For the latter, the CAG numbers of samples were permutated independently and repeatedly for each gene and subsequently correlated with the FPKM. Results are only reported for striatum, as preliminary analyses indicated only weak correlation for cortex and liver. The significance of overrepresentation of UPR genes among significantly correlated genes (fdr < 0.01) was assessed using the Fisher’s exact test.

### Identification of differential expression using Gene Set Enrichment Analysis (GSEA)

We performed gene set enrichment analysis (GSEA) ^46^ (RRID:nif-0000-30629) comparing HD-associated expression to wild type or control data to identify differentially expressed genes. As input, we used the above-mentioned UPR gene sets and HD gene expression data. UPR genes were identified as significant when the enrichment score (ES) corresponded to a *fdr* ≤ 0.05 in HD gene expression data sets. For further analysis, we used only the genes present in the ‘UPR core enrichment’ gene sets. Those genes belonged to the leading-edge subsets and contributed the most to the enrichment scores, and are the most differentially expressed among the UPR genes. To visualize these results, we generated Venn diagrams using jvenn ^47^, to display the common genes across alternative comparisons.

### Identification of stress response elements in the promoter regions

In order to verify the presence of unfolded protein response element (UPRE) and ER stress response elements (ERSE I and II) ^48^ in the upstream regions (-1000bp to +500bp) of UPR genes upregulated in HD, we downloaded all the human promoter regions, (n=23322) available in the eukaryotic promoter database (EDP; http://epd.vital-it.ch) ^49^ (RRID:nif-0000-02806). Next, we used Regulatory Sequence Analysis Tools (RSAT) ^50^ to map these elements in the promoters and computed the enrichment of these stress elements in promoters of upregulated UPR genes compared to all the human promoters using hypergeometric test (equivalent to Fisher’s exact test).

### Functional enrichment analysis

To identify enriched biological processes in our gene sets, we used BiNGO ^51^ (RRID:nlx_149196) for Cytoscape ^52^ ( http://apps.cytoscape.org/apps/bingo) (RRID:nif-0000-30404); and GSEA ^46^ (RRID:nif-0000-30629) to evaluate if genes from curated Reactome pathways (obtained from the Molecular Signature Database (MsigDB) ^53^) were statistically over-represented. The significance of each identified biological process or pathway was calculated using the hypergeometric test, adjusted for multiple testing and converted to *fdr*s using the Benjamini and Hochberg method ^54^ implemented in BiNGO (RRID:nlx_149196) or in GSEA (RRID:nif-0000-30629), respectively. We considered only those processes and pathways with an *fdr* of ≤ 0.05 to be significantly enriched.

### Development of a web-portal for interactive analysis of UPR-associated gene expression

To enable the visualization and interactive inspection of expression of UPR genes across the integrated experiments, we implemented the UPR-HD tool. It is database with a web-interface based on a modified and enhanced version of the GeneXplorer software ^55^. UPR-HD enables the query of individual or multiple UPR genes, the visualization of the expression changes observed in the microarray experiments and their export in table format for further usage. A help page gives an overview of the web-tool’s functionalities. UPR-HD can be freely accessed at http://uprhd.sysbiolab.eu.

## Results and discussion

### Identification of common UPR genes across HD gene expression studies

**Supplementary Figure 1.**
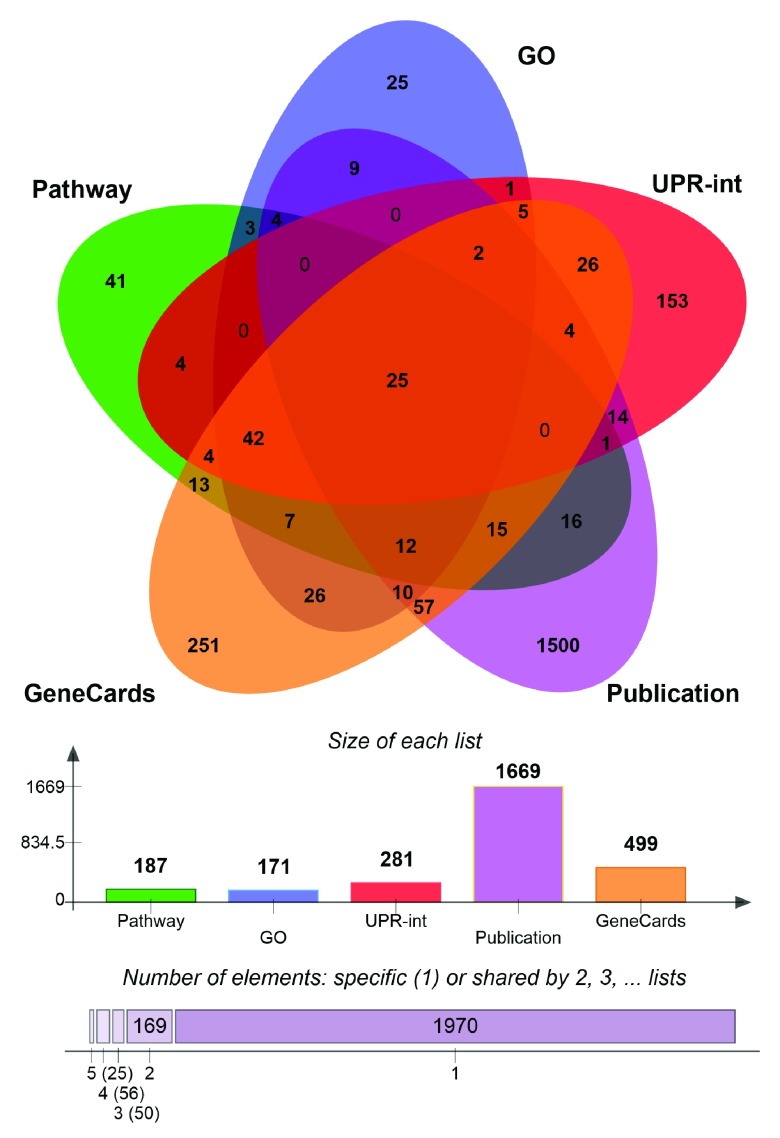
Comparison of UPR gene lists. Venn diagram comparing the three different UPR genes lists generated.

To determine possible implications of the UPR in HD, we sought to assess its activation using a computational approach and the evaluation of existing data. First, we catalogued genes that are involved in UPR from several different sources and divided them into three different categories: UPR-KEGG-GO, UPR-Interactions and UPR-Literature as described in Material and Methods section and detailed in Table 1 and Supplementary Figure 1.

Since changes in gene transcription are main effects of UPR activation and published microarray data are available for HD in humans as well as for HD models, we collected 12 different gene expression datasets generated for the study of HD: three datasets included expression from human blood and brain samples as well as human induced pluripotent stem cells (iPSCs); seven datasets were derived from HD mouse models and cell cultures; one from rat cells and one from yeast cells. If the expression dataset constituted time-series, we split the dataset according to the time points to maintain the temporal aspect of the expression changes.

We reasoned that UPR activity should be reflected in the regulation of UPR genes. By applying GSEA we tested whether UPR genes tend to be differentially expressed in HD samples or models compared to the corresponding controls. GSEA was employed, since it is able to detect modest but consistent tendencies in expression change within a pre-defined set of genes. This can be seen as a crucial advantage, as only small changes in gene expression are frequently observed in the study of neurodegenerative disease due heterogeneity of tissue samples and biological variability of the underlying processes.

**Figure 2.**
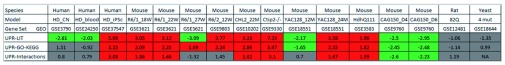
Differential regulation of UPR gene sets detected in HD expression data. For each of the compiled UPR genes sets, the normalized enrichment scores (NES) produced by GSEA are shown for different comparisons of HD-associated expression with controls. Positive scores indicate a tendency towards upregulation; negatives scores indicate a tendency towards downregulation of genes in the UPR sets. Comparisons that showed significant upregulation of UPR gene sets ( *fdr* ≤ 0.05 and NES ≥ +1.4) are highlighted by red background, while significant downregulation ( *fdr* ≤ 0.05 and NES ≤ +1.4) by green background.

Remarkably, we found both indications for significant upregulation as well as repression of UPR genes in the different comparisons. Notably, significant differential expression was generally consistent across the three alternative UPR gene sets (with the exception of R6/1 mice at 27 weeks, where UPR-Literature genes tended to be downregulated while UPR-GO-KEGG genes displayed upregulation) ( Figure 2). This observation implies that the obtained findings tend to be independent of the particular definition of UPR genes chosen in this study.

For most murine *in vivo* HD models, a significant upregulation was detected. Interestingly, the activation pattern was dependent on the time point of expression measurement for the two mouse models (R6/1 and YAC128), for which time series data were available. In the case of R6/1 mice, expressing exon 1 of the human *HTT* gene with a 115 CAG repeat, the most significant activation of UPR genes occurs after 18 and 22 weeks, while genes included in UPR-Literature and UPR-Interactions sets tend to be downregulated after 27 weeks. In contrast, for YAC128 mice containing the full length human *HTT* gene with 128 CAG repeats, upregulation of UPR genes is only observed at the later time point (24 months), whereas downregulation dominates after 12 months. This divergence may be explained by the rapid development of an aggressive disease phenotype in R6/1 mice compared to YAC128 mice, which show a milder phenotype with slower progression ( Figure 2).

Inspecting the three human expression datasets, only the iPSC HD model showed a highly significant activation for all three UPR gene sets, whereas no consistent differential expression of the three UPR gene sets could be detected in blood and brain samples of HD patients. For whole blood samples, this observation might not be surprising, since erythrocytes - constituting the main component of blood - lack of an ER. The absence of a clear pattern in HD brain expression might be due to the fact that expression data were obtained from the post-mortem samples and thus represent typically only the very late stage of the disease. Finally, no significant alterations of expression were found for the rat *in vitro* and the yeast HD model ( Figure 2).

Strikingly, expression of UPR genes also tended to display a strong correlation with the length of the polyglutamine tract in mice. Altogether, the expression of 10.6% of genes was found to be significantly positively correlated, while 7.8% were negatively correlated with the number of CAG. More specifically, we obtained p-values of 1.7∙10 ^-5^, 7.7∙10 ^-6^ and 2.2∙10 ^-16^ for the overrepresentation of correlated UPR-KEGG-GO, UPR-Interactions and UPR-Literature genes, and p-values of 0.10, 3.9∙10 ^-5^ and 4.6∙10 ^-15^ for the overrepresentation of anti-correlated genes, respectively.

**Figure 3.**
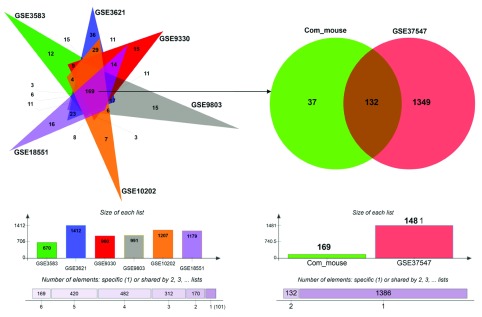
Up regulated UPR genes. Genes included in the core enrichment set for comparisons that indicated UPR activation (highlighted in red in Figure 2) were compared. Common upregulated UPR genes (n=169) in six HD murine models (left side) were intersected with upregulated UPR genes in human HD iPSCs (right side) resulting in a set of 132 UPR genes, whose activation was conserved across the different HD models. The bar plots (bottom) display the number of UPR genes assigned to the core enrichment sets for comparisons that indicated upregulation.

Next, we sought to identify UPR genes which showed conserved differential regulation in distinct HD models. For this purpose, we collected genes that were assigned to the enrichment core by GSEA in the comparisons that showed significant upregulation of UPR genes. In total we collected the UPR genes contained in the enrichment cores from six murine HD models (including one cell line) and the human HD iPSC model. Intersection of these sets led to the identification of 132 genes that were commonly upregulated, when UPR activation was indicated ( Figure 3, Supplementary data file 1). We refer to this set of genes as *UPR-HD ^up^*.

**Supplementary Figure 2.**
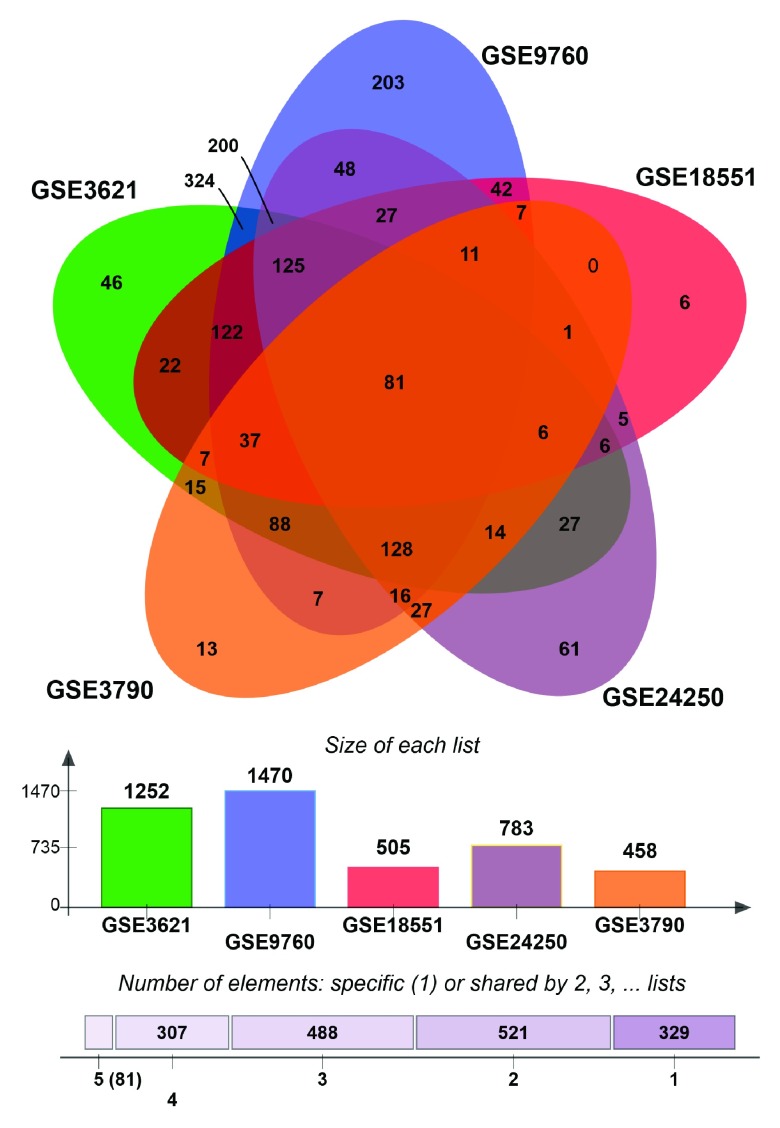
Common downregulated UPR genes. Comparison of several HD expression data sets to identify UPR genes that are downregulated in different HD models.

As the UPR has also been associated with suppression of gene transcription and the enhanced degradation of numerous transcripts ^56, 57^, we carried out the equivalent procedure to identify UPR genes whose downregulation is conserved in different HD models. Here, we intersected the enrichments core from five comparisons displaying suppression of UPR genes ( Supplementary Figure 2, Supplementary data file 2). This resulted in 81 commonly downregulated genes. We refer to the combined set of UPR genes (consisting of 132 upregulated and 81 downregulated genes) that were commonly detected as differentially regulated in HD gene expression data as *UPR-HD ^diff^*.

To capture a larger number of dysregulated UPR genes, we generate alternative gene lists with less stringent requirement for inclusion. In particular, we derived 876 upregulated genes by requiring that they needed to be included in the enrichment cores of the human iPSC and only four out of six murine HD models ( Supplementary data file 1). Similarly, a less stringent list was generated with 388 genes showing downregulation in at least four comparisons ( Supplementary data file 2).

### Examining stress response elements in the promoter regions of upregulated UPR genes

To assess whether the observed upregulation indeed reflects the activation of UPR or if it might be a consequence of other unrelated processes, we carried out an analysis of the promoter regions of genes included in UPR-HD ^up^. We searched for the presence of sequence elements that indicate binding of transcription factors associated with the UPR. In particular, we searched for unfolded protein response elements (UPRE; TGACGTG (G/A)) and the alternative ER stress response elements I (ERSE I; CCAAT(N9)CCACG) and II (ERSE II; ATTGG-N-CCACG) in promoter regions (-1000 bp to +500 bp) regions of UPR-HD ^up^ genes. These characteristic sequence elements are targeted by the bZIP transcription factors ATF6 and XBP1, which are main mediators of the transcriptional adaptation evoked by UPR ^58^.

**Supplementary Figure 3.**
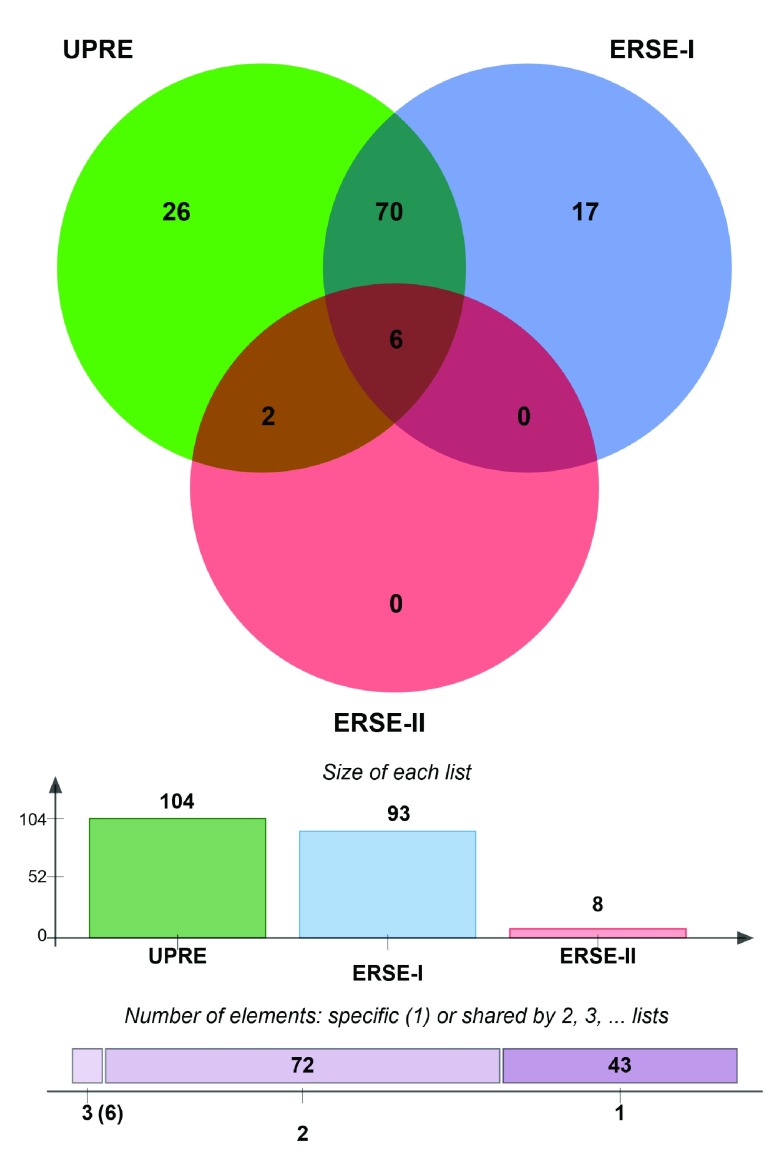
Comparison of UPRE and ERSE elements in the promoter region of UPR genes that are upregulated in HD.

Strikingly, we found that the vast majority of the UPR-HD ^up^ has such a characteristic binding sequence in their promoter regions ( Supplementary Figure 3). Compared to number of sequence elements that we would expect by chance, a highly significant overrepresentation was detected for the UPR-HD ^up^ genes. More specifically, we found the occurrence of UPRE in 104 genes ( *p*=3.0∙10 ^-9^), ERSE-I in 93 genes ( *p=0.0019*), and ERSE-II in 8 ( *p=0.052*). Notably, a large number of UPR-HD ^up^ genes had alternative binding motifs included in the promoter region: 70 genes had both ERSE-I and UPRE, 2 genes had both ERSE-I and ERSE-II and six genes had all three elements (for list of genes see Supplementary data file 3) which might suggest that these genes are under particularly tight control of UPR-associated transcription factors ATF6 and XBP1. We also detected a highly significant enrichment for the less stringent list of upregulated UPR genes. Here, we found that 684 genes have UPRE ( *p*=4.4∙10 ^-41^), 562 have ERSE-I ( *p*=4.4∙10 ^-5^) and 56 have ERSE-II ( *p*=3.8∙10 ^-5^) in their promoter region ( Supplementary data file 3). Altogether, the results of the promoter analysis support the conclusion that the upregulation of UPR genes in HD models faithfully reflects an activated UPR.

### Biological processes that are enriched in differentially expressed UPR genes

**Figure 4.**
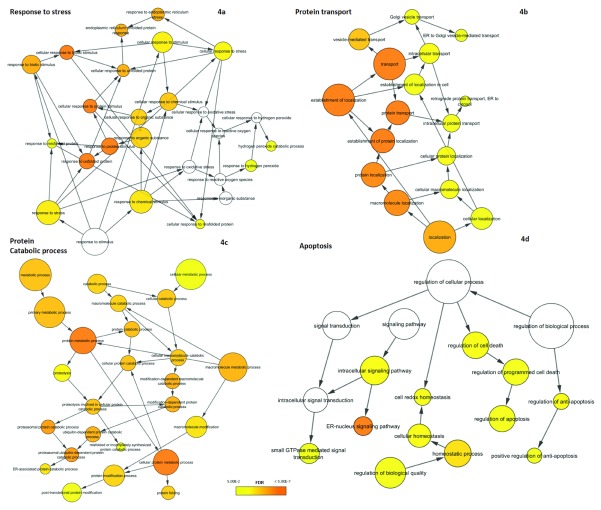
Biological processes enriched among differentially regulated UPR genes. GO hierarchies for biological processes overrepresented in the set of differentially regulated UPR are shown. Nodes indicate specific GO terms and their size represents the number of included UPR genes. The significance of overrepresentation (enrichment) is visualized by colour-coding from yellow to orange with the latter representing higher significance. No colour indicates that the process is not significantly enriched ( *fdr* ≥ 0.05). The overrepresented biological processes can be split into four major themes: ( **a**) response to stress, ( **b**) protein transport, ( **c**) protein catabolic process and ( **d**) apoptosis.

Since the UPR comprises a complex series of diverse molecular mechanisms, we examined the functional composition of UPR-HD ^diff^ genes. For this purpose, we performed functional analysis using BiNGO to identify enriched biological processes (as defined in GO) that are overrepresented among UPR-HD ^diff^ genes. All the biological processes that are significantly enriched in our analysis are listed in Supplementary data file 4 as well as those for the less stringent list of differentially regulated UPR genes. Expectedly, we detected that stress-related functional categories such as ‘response to stress’ (GO ID:6950; n=44; *fdr*=2.08E-03) and ‘response to unfolded protein’ (GO ID:6986; n=13; *fdr*=4.55E-09) were enriched ( Figure 4a). A second group of significantly overrepresented GO categories were related to ‘protein transport’ (GO ID:15031; n=34; *fdr*=1.76E-07) and ‘protein localization’ (GO ID:8104; n=36; *fdr*=1.19E-06) including ‘vesicle-mediated transport’ (GO ID:16192; n=24; *fdr*=9.03E-05) and ‘ER to Golgi vesicle-mediated transport’ (GO ID:6888; n=4; *fdr*=4.00E-02) ( Figure 4b). Additionally, we also found ‘ER-nucleus signalling pathway’ (GO ID:6984; n=9; adjp-value=1.81E-07) to be highly enriched. It has been previously reported that ER-nucleus signalling pathway functions via activation of NF-κB due to ER-overload triggered by protein congestion ^59^.

Furthermore, UPR-HD ^diff^ genes tended to be associated with protein catabolism and in particular protein degradation ( Figure 4c). Significant processes here were e.g. ‘protein catabolic process’ (GO ID:30163; n=18; *fdr*=6.40E-05), ‘proteasomal ubiquitin-dependent protein catabolic process’ (GO ID:43161; n=13; *fdr*=6.69E-06) and ‘protein ubiquitination’ (GO ID:31396; n=7; *fdr*=3.12E-02). These results coincide well with previous studies establishing the connection of UPR and ERAD and showing, for instance, that the extent of activation of the UPR is concurrent with the severity of ERAD defect ^60^.

Finally, genes linked to apoptosis could be found among the UPR-HD ^diff^ genes ( Figure 4d). Of particular interest for potential intervention could be genes associated with ‘regulation of apoptosis’ (GO ID:42981; n=24; *fdr*=1.65E-02), as their manipulation may prevent the execution of the apoptotic programme under persistent ER stress.

In summary, UPR genes detected as commonly differentially regulated in HD expression data were not restricted to a particular functional category, but can be associated with many processes linked to the UPR.

### Pathways enriched in upregulated genes

**Figure 5.**
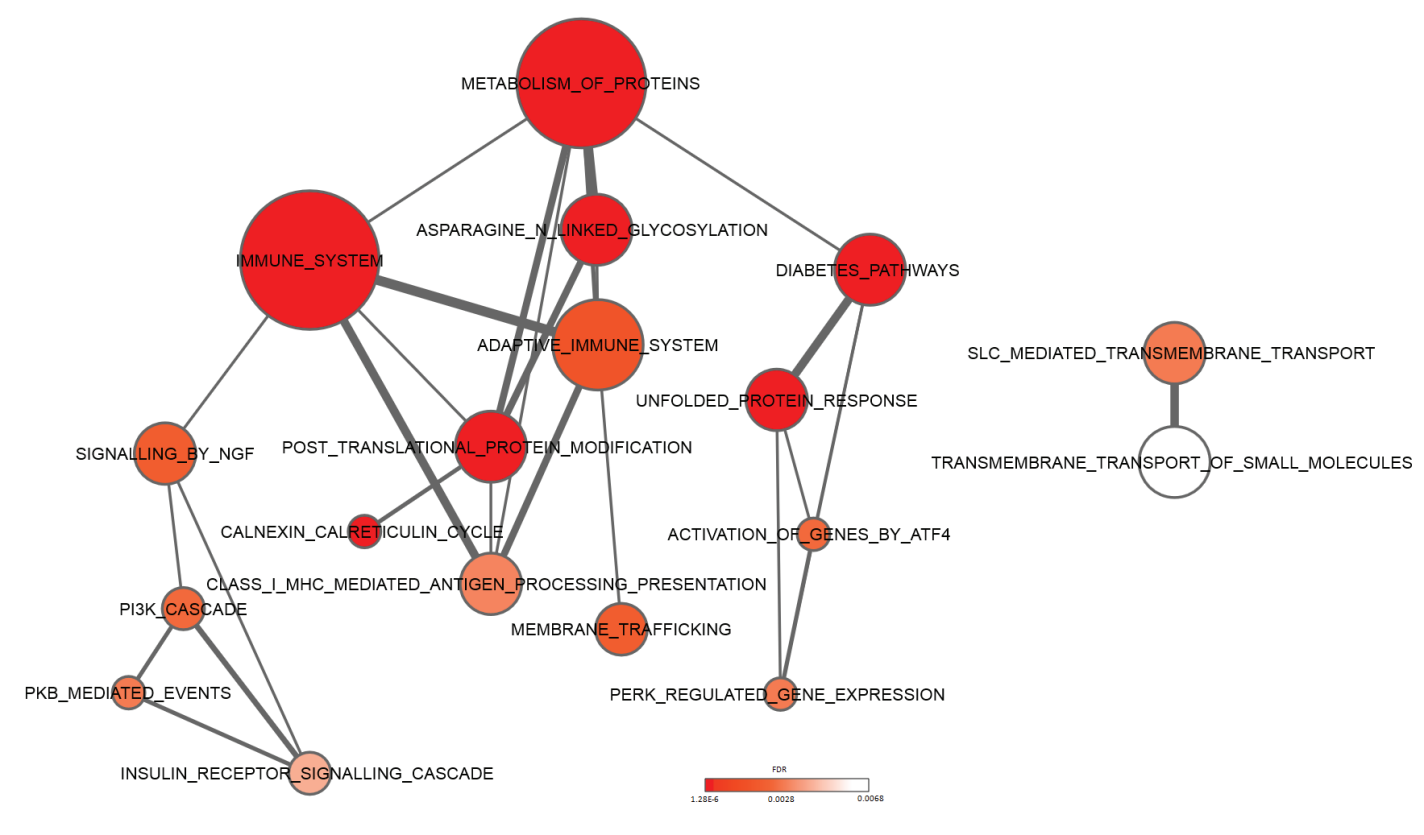
Pathways enriched among upregulated UPR genes. The network of pathways with overrepresentation of upregulated UPR genes are shown. Node size represents number of genes in each pathway and connecting line size represents number of genes shared between two pathways. Colour coding from white to red indicates increasing statistical significance. Pathways were derived from the Reactome database.

Complementary to the functional composition, we evaluated whether specific pathways might be activated based on the observed commonly upregulated UPR genes (UPR-HD ^up^). Therefore, we carried out pathway enrichment analysis using a set of pathways curated in the Reactome database. As expected, ‘unfolded protein response’ (n=6; *fdr*=2.04E-05), ‘activation of genes by ATF4’ (n=3; *fdr*=0.00291) and ‘PERK regulated gene expression’ (n=3; *fdr*=0.0033) were detected as significantly enriched among UPR-HD ^up^ genes ( Figure 5). More interestingly, we also found an overrepresentation of components of the ‘immune system’ (n=14; *fdr*=6.74E-05), ‘adaptive immune system’ (n=6; *fdr*=1.96E-03), ‘NGF signalling’ (n=9; *fdr*=2.42E-03), and ‘Diabetes pathways’ (n=7; *fdr*=2.04E-05). Complete results from the analysis are included in Supplementary data file 5.

Remarkably, recent studies have also suggested that ER stress and activated UPR are interconnected with inflammatory processes ^61^. Inflammation is an immunological process usually carried out by the vascular system to counteract disease, and to fight foreign antigens against invasion. Within the brain, microglia and astrocytes play important immunological functions. Until very recently, little was known about inflammatory molecules in HD. Recent studies, however revealed a distinct profile of inflammatory mediators from post-mortem human HD tissue ^62, 63^. Inflammatory mediators such as IL-1β and TNF-α were increased only in the striatum, whereas IL-6, IL-8 and MMP-9 were also upregulated in cortex and in the cerebellum ^63^. This supports the conjecture that secreted inflammatory cytokines and activated microglia cells could lead to axonal damage and extensive neuronal cell death in HD pathology ^62– 65^. In general, activated microglia exert their diverse effects on neurons and macroglia (astrocytes and oligodendrocytes). Inflammation occurs through the release of cytoprotective agents such as growth factors, plasminogen, and neuroprotective cytokine as well as cytotoxic substances such as oxygen radicals, nitricoxide, glutamate, proteases, and neurotoxic cytokines. One of the earliest reports describing microglial abnormalities in HD was provided by Singhrao *et al.* ^65^. Microglial cell counts were considerably increased in the caudate putamen of HD and these microglial cells expressed increased amounts of complement factors. A more detailed investigation of microglial morphological changes associated with HD was performed by Sapp *et al.* ^66^. The authors localized morphologically activated microglial cells in the neostriatum, cortex and globus pallidus as well as in adjoining white matter of HD brains. Additionally, positron emission tomography (PET) studies using the ligand for benzodiazepine receptor (PK-11195), which labels activated microglia have been employed to study of neuroinflammation. Using this technique, Tai *et al.* ^67, 68^ demonstrated that microglial activation in HD patients correlates with disease progression as assessed by loss of dopamine D2 receptor binding sites. Interestingly, Tai *et al.* could also demonstrate that microglial activation and release of cytokine IL-6 can be observed in presymptomatic HD gene carriers and can be detected up to 15 years before predicted age of onset. These findings indicate the microglial inflammatory activation is an early event associated with subclinical progression of HD and may constitute a target for early therapeutic intervention.

Besides the indication of processes related to the immune response, results of the pathway enrichment analysis also pointed to diabetes. It has been shown that diabetes in Wolcott-Rallison syndrome (a rare autosomal recessive form of juvenile diabetes) is a result of high levels of ER stress caused by mutations in the PERK gene in pancreatic β-cells. In addition, studies have shown that HD patients show increased incidence of diabetes ^69, 70^ and HD transgenic mice develop hyperglycemia ^71, 72^. More recently it has been experimentally validated that HD transgenic mice develop intranuclear inclusions in the pancreatic β-cells, causing an intrinsic defect in insulin production ^73^.

For the enlarged list of upregulated UPR genes, additional pathways were detected to be significantly enriched ( Supplementary data file 5). For instance, genes associated with cell cycle and apoptosis were strongly overrepresented among the upregulated UPR genes.

### Prioritization of UPR-HD connectors through integrative analysis

To narrow down the list of UPR-HD ^diff^ genes for further inspection, we utilized additional information, including a reference set of potential molecular targets for HD therapy that were made available through the HD Research Crossroads database initiated by the CHDI Foundation (see Kalathur *et al.* 2012) ^41^. Genes were included by experts in the field based on the evaluation of published literature and in-house screens using a set of defined criteria (see Kalathur *et al.* 2012) ^41^. For instance, a gene was considered as a potential HDTT if genetic or pharmacologic modification of its activity led to a change of a HD-related phenotype in a validated cell culture or organism model of HD. At present, this curated reference set constitutes the most comprehensive collection of HDTTs. In addition, we extracted genes, whose corresponding proteins were reported to be physically associated with HTT, from the HDNetDB database. We recently demonstrated that HTT interactors tend to be enriched in proteins that influence the toxicity of mHTT, and provide favourable candidates for the identification of molecular modifiers of HD ^74^.

**Supplementary Figure 4.**
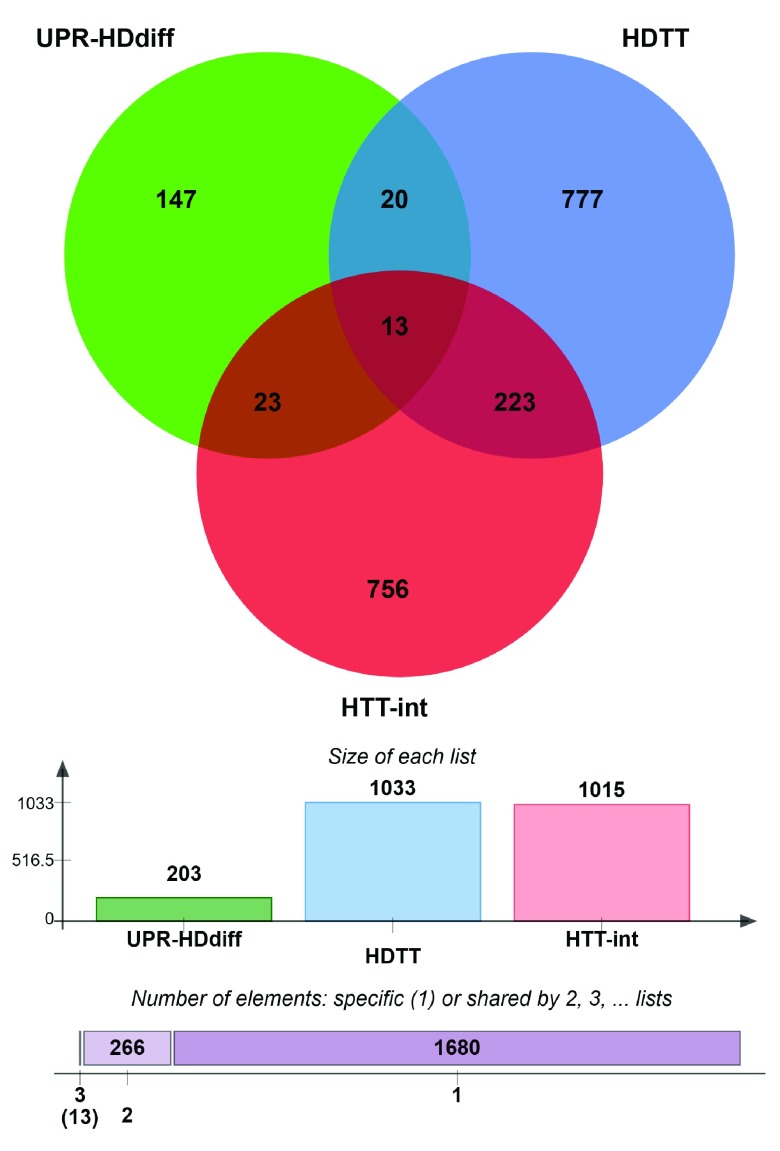
UPR-HD connectors. Venn diagram showing common genes between 3 data sets, high stringency UPR-HD ^diff^: UPR genes that are differentially regulated in HD i.e. either upregulated in human HD iPSC and 6 murine HD models or downregulated in 5 murine HD models; HDTT: HD therapeutic targets as described by Kalathur RK *et al.* ^41^ and originated by the HDCrossRoads database; and HTT-int: HTT interactors derived from the HDNetDB.

We reasoned that differentially regulated UPR genes, which have been shown to influence HD-related phenotypes and to be physically associated with HTT, could constitute molecular links between UPR and HD. Therefore, we integrated the three gene lists and identified 13 genes that were common to all three: *RAB5A, HMGB1, CTNNB1, DNM1, TCP1, TUBB, TSG101, DNAJB1, CCT2, EEF2, DYNC1H1, HSPA5* and *SLC12A5* ( Supplementary Figure 4). Most of the corresponding proteins are indirect interactors of HTT, while two ( *CTNNB1, DNM1*) interact directly ( Table 3, Supplementary Figure 4). Notably, the search for stress response elements in the upstream regions of these 13 genes revealed that eight genes ( *RAB5A, HMGB1, CTNNB1, DNM1, TCP1, TUBB, TSG101* and *DNAJB1*) possess either UPRE or ERSE or both elements in their promoters, suggesting that these genes are under direct control of UPR-associated transcription factors ( Table 3). Moreover, the expression of the 13 gene tends to be strongly correlated with the length of the polyglutamine tract in HD mice. Indeed, 9 of the 13 genes were found to be significantly correlated, whereas we would expect only 1.4 by chance.

**Table 3.** Differentially regulated UPR genes that interact with HTT and were classified as potential HD therapeutic targets (HDTT). + indicates the presence of ER stress-associated sequence motifs (UPRE, ERSE-I, ERSE-II) in the promoter regions (+1000 to -500 bp). Interaction type indicates whether the protein was shown to have a direct or indirect physical interaction. The Spearman correlation coefficient and the corresponding estimated fdr describe the correlation of gene expression with the length of polyglutamine tract in HD mice.

Gene	UPRE	ERSE-I	ERSE-II	Interaction type	CAG correlation coefficient	fdr cor.
RAB5A	+	+		Indirect	0.48	0.0038
HMGB1	+	+		Indirect	0.39	0.026
CTNNB1		+		Direct	0.82	0
DNM1	+	+		Direct	0.55	0.00050
TCP1	+	+		Indirect	0.62	0.00014
TUBB	+	+		Indirect	0.60	0.00025
TSG101	+	+		Indirect	0.73	0
DNAJB1	+		+	Indirect	-0.22	1
CCT2				Indirect	0.79	0
EEF2				Indirect	0.81	0
DYNC1H1				Indirect	0.57	0.00038
HSPA5				Indirect	0.07	0.81
SLC12A5				Indirect	-0.06	1

Inspection of the genes possessing UPRE or ERSE elements in their promoter regions revealed that four of them ( *TCP1, CCT2, DNAJB1 and HMGB1*) have been reported to act as chaperones. Besides being essential components of the UPR, molecular chaperones can modulate the aggregation and toxicity of proteins, including mHTT. TCP1 (CCT1) and CCT2 are components of the TCP1 ring complex (TRiC) that uses cycles of ATP-binding and hydrolysis to bind unfolded polypeptides and facilitate their folding. Notably, TRiC has been identified as a potent suppressor of mHTT mediated toxicity and inhibitor of the mHTT protein aggregation *in vitro* and *in vivo* ^75^. DNAJB1 belongs to the group of DnaJ/Hsp40 (Heat shock protein 40) proteins that are involved in protein translation, folding and translocation through regulating ATPase activity of the Hsp70s chaperones. In a PC12 cell model, experiments indicated that DNAJB1 attaches to soluble mHTT oligomers and recruits Hsp70 suppressing mHTT mediated toxicity ^76^. Finally, *HMGB1* encodes for the High-mobility group box 1 protein (HMGB1), which has recently been demonstrated to have chaperone-like activity, inhibiting aggregation of various proteins. Overexpression of HMGB1 can also decrease the aggregation induced by extended polyQ stretches ^77^.

Intersecting the less stringent list of differentially regulated UPR genes with HTT interactors and HDTT led to the identification of additional 40 genes that might link UPR and HD ( Supplementary dataset 9). Of particular interest might be 15 genes, whose corresponding proteins are direct interactors of HTT. They include ubiquilin 1 ( *UBQLN1*), the ubiquitin ligase synoviolin ( *SYVN1*) involved in ERAD and the ubiquitin ligase *ATG5*, which is necessary for autophagy, as well as the initator ( *CASP2*) and effector ( *CASP7*) caspases. Moreover, the major molecular regulators such as *TP53*, *AKT1* and *SP1* are members of this candidate set.

### Linking the UPR network to apoptosis and HTT

A crucial aspect of the UPR in the context of HD is the possibility that it can trigger apoptosis upon persistent ER stress. To obtain a comprehensive view of the connections between UPR and apoptosis, we applied a network approach. First, we generated the UPR interactome from known protein interactions of UPR core components, which we extracted from the UniHI and HDNetDB databases ( Supplementary data file 6). Second, we compiled a list of genes (n=594) associated with apoptosis from several different sources (as described in the materials and methods). We then used this list to identify 40 proteins associated with apoptosis within the UPR interactome ( Supplementary data file 7). These genes included, among others, Apoptosis Signal Regulating Kinase 1 (ASK1, also known as MAP3K5), whose knock-out in primary neuron provided protection from ER stress-induced JNK activation and cell death triggered by polyQ fragments ^78^.

**Figure 6.**
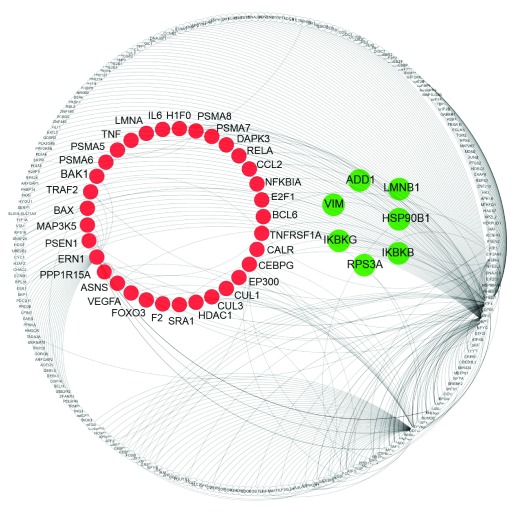
Network representation of UPR-apoptosis connection. The network displays UPR proteins and their interactions. Nodes indicate proteins and lines represent molecular interactions between them as derived from UniHI and HDNetDB. UPR proteins which are also associated with apoptosis are highlighted in red, while UPR proteins that are both associated with apoptosis and identified as HTT interactors are in green.

As the mutation in HTT can perturb the function of interacting proteins by aberrant binding, we checked for each of the 40 proteins whether they have been reported to physically associate with HTT. Using molecular interaction data collected in HDNetDB, we detected that six of the 40 proteins interact with HTT i.e. ADD1, HSP90B1, IKBKB, RPS3A, IKBKG and LMNB1 ( Supplementary data file 7). A visualisation of the UPR interactome with apoptosis-related proteins and HTT interactors highlighted can be found in Figure 6.

Literature review showed that the two proteins kinases IKBKB and IKBKG, the laminin LMNB1 and the ribosomal protein RPS3A have been previously linked to neurodegenerative diseases. IKBKB and IKBKG are subunits of IkB kinase (IKK). They activate members of the NF-κB transcription factor family by phosphorylation of their inhibitor (IkB) ^79^ leading to ubiquitination and destruction of IkB, thereby allowing activation of the NF-κB complex. NF-κB maintains the balance between cell survival and apoptosis ^80^. Although unrelated to ER stress, it has been shown that inhibition of IKBKB decreases HTT proteolysis in a cell model, and thus might lower the load of toxic HTT fragments in HD ^81^. Recently, it has been reported that ubiquitination of IKBKG by Parkin, an ubiquitin ligase associated with Parkinson’s disease regulates the anti-apoptotic pathway that is key to maintaining mitochondrial integrity ^82^.

Lamin B1 protein, LMNB1 is thought to be involved in nuclear stability and chromatin structure. Experiments in *Caenorhabditis elegans* overexpressing aggregation-prone peptides identified laminins as modulators of protein toxicity at neuromuscular junctions ^83^. Further, in leukodystrophy mouse models, lamin B1 acts as an important regulator of myelin formation and maintenance ^84^, while in humans lamin B1 gene duplications ^85^ and large deletions upstream of promoter regions can cause autosomal-dominant leukodystrophy ^86^. More importantly, a recent study reports increased levels of lamin B1 in both human HD patients and the R6/1 mouse model of HD ^87^. Due to the involvement of lamin B1 in several cellular alterations such as chromatin organisation, gene transcription and proteotoxicity, alterations in lamin B1 expression might have important implications in HD pathophysiology.

Finally, it has been demonstrated that apoptosis is induced by inhibiting the expression of ribosomal protein S3A (RPS3A) ^88^. It also has been observed that SNP variants in RPS3A homologues are associated with pathogenesis of Alzheimer’s disease ^89^. Apart from its function as a ribosomal protein, RPS3A might also act as a chaperone. Co-expression of mouse RPS3A suppressed the toxicity induced by α-synuclein (which is a major components of Lewy bodies observed in Parkinson’s disease) in a yeast model system ^90^.

As the literature review indicated, the intersection of the UPR interactome with apoptosis-related genes and HTT interactors can point out proteins with potential relevance for neurodegeneration. Thus, the generated gene lists provided in the Supplementary data file 6 and Supplementary data file 7 might give interested researchers a valuable basis for more detailed inspections.

Raw data for Kalathur et al., 2015 ‘The unfolded protein response and its potential role in Huntington ´s disease elucidated by a systems biology approach’ Data file 1. Legend: Lists of upregulated genes in 6 murine and 1 human (first sheet), and in at least 4 murine and 1 human HD model (second sheet).
Data file 2. Lists of downregulated genes in 5 datasets (first sheet) and in at least 4 datasets (second sheet).
Data file 3. Lists of upregulated UPR genes with stress response elements in the promoter regions (-1000bp to +500bp). Results for highly stringent gene list (UPR genes uprgulated in 6 murine and 1 human HD model) are show on sheet 1; results for less stringent gene list (upregulated in at least 4 murine and 1 human HD model) are displayed on sheet 2.
Data file 4. List of all the biological processes that are enriched amongin differentially regulated UPR genes. Results for UPR gene list of high stringency are show on sheet 1; results for gene list of lower stringency are displayed on sheet 2.
Data file 5. List of all the pathways that are enriched among upregulated UPR genes. Results for UPR gene list of high stringency are show on sheet 1; results for gene list of lower stringency are displayed on sheet 2.
Data file 6. List of components of UPR interactome shown in Figure 6
Data file 7. List of genes common between UPR and Apoptosis
Data file 8. List of proteins that interact with HTT directly or indirectly.
Data file 9. List of 53 proteins that were found in the intersection of HTT interactors, HDTT and differentially regulated UPR genes (of lower stringeny). It includes the 13 proteins from table 3 that were identified using the high stringency list of differentially regulated UPR genes. Spearman correlation coefficients and the corresponding estimated fdr for correlation or anti correlation, respectively, describe the observed correlation of gene expression and the length of polyglutamine tract in HD mice. Click here for additional data file. Copyright: © 2016 Kalathur RKR et al. 2016 Data associated with the article are available under the terms of the Creative Commons Zero "No rights reserved" data waiver (CC0 1.0 Public domain dedication).

## Conclusions

Various studies have indicated a role of the UPR in HD. However, its relevance for therapeutic interventions remains to be established. With the presented work, we aimed to delineate the connection between UPR and HD by examining available HD-relevant gene expression and molecular interaction data. We found indications for differential regulation of UPR genes in a number of expression studies. Notably, the observed differential regulation is not conserved across all evaluated studies reflecting the well-known heterogeneity of current HD models. This needs to be taken into account for future studies of the UPR in the context of HD. The results of our analysis (displayed in Figure 2) may therefore serve as guidance for the choice of model systems. Despite the observed heterogeneity, the comparison nevertheless indicated a number of genes that tend to be commonly regulated in different expression studies. This finding enabled us to define core sets of UPR genes that were commonly up- or downregulated in different studies. This derivation was supported by the detection of a significant overrepresentation of UPR-associated stress response elements (UPRE and ERSE) in the promoter regions of the upregulated genes. Moreover, we observed that expression of UPR genes in the striatum tend to correlate with the length of the HTT polyglutamine tract in mice. This is a remarkable observation, since human patients with longer polyglutamine tracts frequently display earlier age of disease onset and death. The observed strong correlation suggests that the UPR might contribute to dynamics of HD, and thus might present a prime target for inventions aiming to delay the appearance of symptoms and to decelerate disease progression.

Functional enrichment analysis on differentially expressed UPR genes pointed to a broad range of mechanisms involved. Additional pathway analyses indicated the activation of inflammatory processes and a potential connection to diabetes. Including complementary data sets, we identified UPR genes that have been indicated to influence HD pathogenesis. Finally, we derived sets of genes that connect UPR with apoptosis and might be directly influenced by mHTT.

In summary, through our work we present the first comprehensive analysis of UPR activation in HD and elucidate potential links to pathogenetic mechanisms within a systems biology framework. While our work cannot provide definite proofs for the identified relations due to its purely computational nature, it can nevertheless constitute a broad basis for experimental follow-up investigations. To assist such endeavours, extensive Supplementary material has been provided together with this article with the aim of helping independent researchers to select genes of interest. We have developed also a publicly accessible web-portal ( http://uprhd.sysbiolab.eu) for the retrieval and visualisation of changes in UPR-associated gene expression across the evaluated transcriptomics studies. In conclusion, we hope that our work can contribute to a better understanding of the UPR in HD and eventually to the identification of novel therapeutic targets to cure HD.

## Data availability

The data referenced by this article are under copyright with the following copyright statement: Copyright: © 2016 Kalathur RKR et al.

Data associated with the article are available under the terms of the Creative Commons Zero "No rights reserved" data waiver (CC0 1.0 Public domain dedication). 

*Figshare*: Raw data for Kalathur *et al.*, 2015 ‘The unfolded protein response and its potential role in Huntington’s disease elucidated by a systems biology approach’ doi: 10.6084/m9.figshare.3080608 ^91^

## Figures and Tables

**Supplementary Figure 5.**
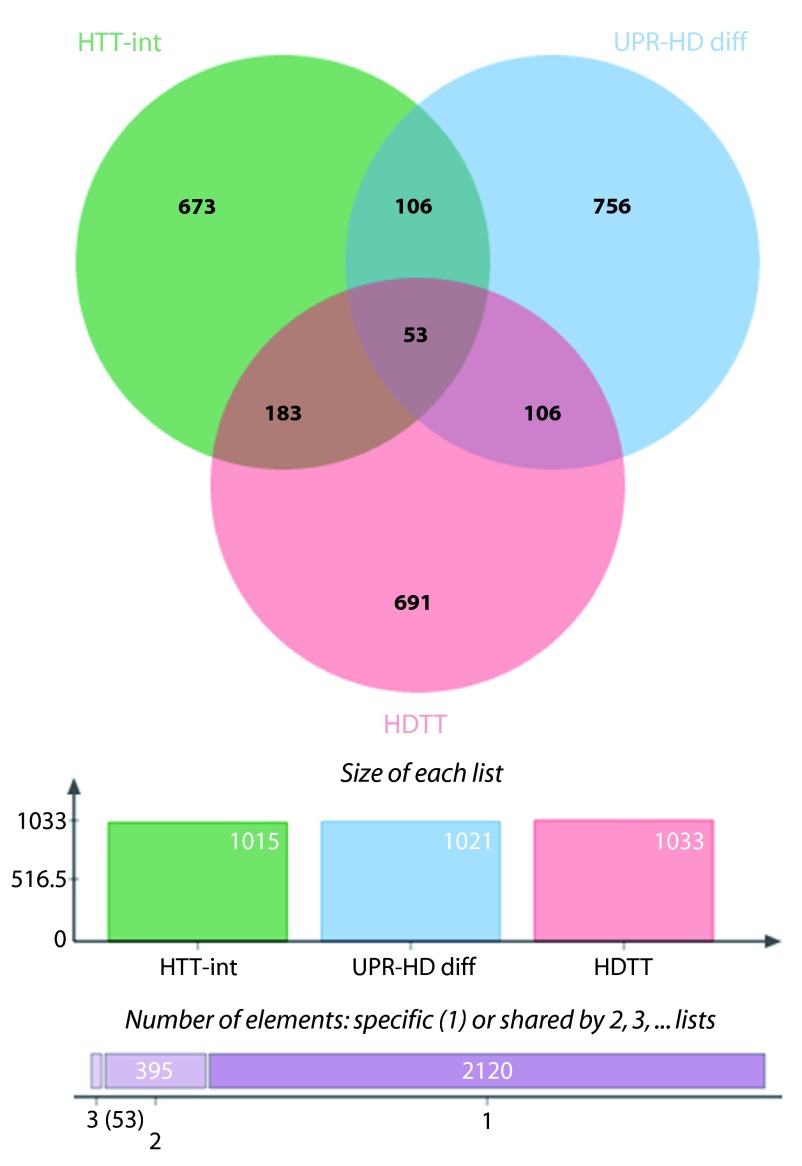
UPR-HD connectors for low stringency UPR-HD ^diff^. Venn diagram showing common genes between 3 data sets, low stringency UPR-HD ^diff^: UPR genes that are differentially regulated in HD i.e. either upregulated in human HD iPSC and at least 4 of 6 murine HD models or downregulated in 4 murine HD models; HDTT: HD therapeutic targets as described by Kalathur RK *et al.* ^41^ and originated by the HDCrossRoads database; and HTT-D-int: direct HTT interactors derived from the HDNetDB.
