# Supplementary material for: The unfolded protein response and its potential role in Huntington's disease elucidated by a systems biology approach
Source: F1000Res. 2016 Mar 2;4:103. Originally published 2015 May 1. [Version 2] doi: 10.12688/f1000research.6358.2 (PMC4758378; doi:10.12688/f1000research.6358.2)
Supplement: Peer review history

# Peer review: Reviewer response for version 2

**DOI:** 10.5256/f1000research.8528.r9522

**Published:** 2016-03-03

**Authors:** Scott Zeitlin

**Affiliations:** 1 Department of Neuroscience, School of Medicine, University of Virginia, Charlottesville, VA, USA

The authors' have added important data showing that changes in UPR gene expression strongly correlate with CAG repeat length.

I confirm that I have read this submission and believe that I have an appropriate level of expertise to confirm that it is of an acceptable scientific standard.

# Peer review: Reviewer response for version 1

**DOI:** 10.5256/f1000research.6818.r9517

**Published:** 2015-08-05

**Authors:** Stefan Taubert

**Affiliations:** 1 Department of Medical Genetics, University of British Columbia, Vancouver, BC, Canada

In this paper, Kalathur *et al. *investigated the role of the ER's unfolded protein response (UPR) in the disease pathogenesis of Huntington's Disease (HD). Although HTT, the protein mutated in HD, does not localize to the ER, mutated HTT intereferes with downstream UPR processes, which may prevent the UPR from functioning normally and thus result in the induction of apoptosis by the UPR, which in turn could underlie the dramatic neuronal loss observed in HD. To broadly evaluate the role of UPR activation in the many experimental HD models and contexts, the authors used a bioinformatics approach to query for the role of the UPR in HD pathology, assessing transcriptomes, regulatory DNA elements, and UPR interactomes. The resulting data point to a core set of UPR genes that were commonly (although, as nicely pointed out, not universally) up- or downregulated in distinct experimental HD models, pro-inflammatory events involving microglia, a putative link between HD and diabetes/hyperglycemia, and the identification of a core set of genes that link UPR to apoptotic signaling and that mHTT may impinge upon.

Overall, this is a nice body of work. This computational analysis is comprehensive and multifaceted, the data interpretation measured and well-qualified, the paper well written, and the data are very accessible. One of the key strengths is that the authors aimed to integrate data obtained in many diferent experimental HD systems - various mouse models, human data, and even yeast and rat models. This comprehensive approach allows them to point to evolutionarily conserved genes and processes as suitable candidates for future investigation.

Specific criticism:

- To derive their set of HTT interacting proteins, the authors use a dataset downloaded from HDNetDB database, which yields a large set of HTT interactors (HTT-int) including 1015 genes, including, as acknowledged, presumably many indirect interactors. Perhaps a deeper up-front curation for putative direct interactors would have been useful.
- In Figure 2, the authors assess the overlap between genes regulated in 6 mouse HD datasets and one human HD. To ensure best stringency, the first limit the mouse dataset to those genes that are regulated in all 6 individual datasets. While this high stringency is laudable, one could have also lowered the stringency a bit in regards to inclusion in the core mouse IPR set, i.e. inclusion in 6/6 sets was deemed required, but expanding the set to 5/6 or even 4/6, resulting in 420 and 902 additional genes, respectively, would have only marginally lowered the stringency while providing a larger set for the determination of evolutionary conservation. 

I confirm that I have read this submission and believe that I have an appropriate level of expertise to confirm that it is of an acceptable scientific standard.

# Response

**Published:** 2016-02-17

**Authors:** Ravi Kiran Reddy Kalathur

**Affiliations:** Centre for Biomedical Research, University of Algarve, Portugal

We thank the reviewer for the positive comments and specific suggestions that helped us to further improve our manuscript. In the revised version, we have included the additional analysis suggest by the reviewer.

1. We distinguish now between direct and indirect interactions of HTT based on their annotation in HDNetDB. An additional supplementary table (Supplementary dataset 8) presents now the classification of HTT interactions. This information was also used for the interpretation of our results. For instance, 2 of the 13 proteins constituting a putative link between HD and UPR, were direct interactors. We did not want to exclude a priori all indirect HTT interactors, since those might be part of larger protein complexes with HTT and thus potentially important for the HTT’s molecular functions.
2. Additional lists of genes with evolutionary conserved expression were generated for less stringent filters (i.e. requiring the inclusion in the enrichment cores of at least 5 or 4 comparisons for up- or downregulated genes, respectively). In general, promotor and enrichment analyses of the extended gene lists agree well with those for the more stringent genes lists. The results are presented in supplementary tables and figures, and briefly discussed in the main manuscript. For the connection to HD, lowering the requirement led to detection of 53 candidates, of which 15 were direct HTT interactors (Supplementary figure 5). This enlarged set is presented in Supplementary dataset 9 for the interested reader.

# Peer review: Reviewer response for version 1

**DOI:** 10.5256/f1000research.6818.r9713

**Published:** 2015-07-29

**Authors:** Scott Zeitlin

**Affiliations:** 1 Department of Neuroscience, School of Medicine, University of Virginia, Charlottesville, VA, USA

In HD and other neurodegenerative disorders involving the accumulation of misfolded protein, ER stress and the activation of UPR have been implicated in pathogenesis, although the extent to which it contributes to neuronal cell death is still unclear. In this paper, the authors employ a bioinformatic approach to show that the unfolded protein response (UPR) is activated in a variety of human and animal Huntington’s disease (HD) models. It is noteworthy that the authors also demonstrate that the upregulation of the UPR genes is most likely a direct response to UPR activation by analyzing the promoter regions of these genes for the binding sites of the UPR transcriptional activators ATF6 and XBP1. Moreover, the authors also identified sets of genes that provide a potential link between both UPR and HD and between UPR and apoptosis. Pathway enrichment analysis was also used to identify functional pathways activated by the common set of upregulated UPR genes. In addition to the expected pathways associated with UPR (e.g. PERK-regulated gene expression and ATF-activated genes), the authors also identified components of the immune system, neurotrophin signaling, and diabetes. Interestingly, there is evidence in the HD literature that all three of these latter pathways are affected in HD.

This work is a good example of the power of using a systems biology approach to provide a fairly comprehensive analysis of UPR activation in HD. The authors are appropriately cautious in emphasizing that their results do not prove that the pathways and relationships between pathways that they have identified all contribute to HD pathogenesis. However, their results provide an excellent guide for further experimental studies, and the authors’ development of a publicly accessible web site for the retrieval and visualization of their UPR-associated gene expression data in HD will be an important tool for the field that will facilitate these future studies.

Minor comments:

1. In the introduction, the authors mention that although HTT is not located in the ER, there are there are several potential mechanisms by which mutant HTT could induce ER stress (impairment of ERAD, dysfunctional vesicular trafficking, and altered ER calcium homeostasis). Atwal *et al., *(2007) and Atwal and Truant (2008) have shown that there is a more direct link between HTT and ER stress.  In their work, they show that the N17 domain of HTT is a stress-sensitive ER association domain, and that the expanded polyQ stretch in mutant HTT perturbs the release of mutant HTT from ER and its translocation of HTT into and out of the nucleus in response to cell stress events.
2. p.7, 1 ^st^ paragraph: “…UPR has been also associated with the suppression or degradation of a substantial number of genes…. Do the authors mean: …UPR has been also associated with the suppression or degradation of a substantial number of genes or gene products?
3. To account for the differences in differential UPR gene expression changes that were observed among the different HD mouse models, the authors suggest that differences in the *HTT* transgenes and their expression levels among the models could be responsible. In addition, the different genetic backgrounds of the models can contribute to the variability. In future studies aimed at studying the role of the length of mutant HTT’s expanded polyQ stretch in UPR activation, the authors may also want to consider evaluating CHDI’s publicly available transcriptome data obtained from the cortex and striatum of 6-month old knock-in HD mouse models expressing wild-type or mutant *Htt* alleles with different CAG repeat lengths.

I confirm that I have read this submission and believe that I have an appropriate level of expertise to confirm that it is of an acceptable scientific standard.

# Response

**Published:** 2016-02-17

**Authors:** Ravi Kiran Reddy Kalathur

**Affiliations:** Centre for Biomedical Research, University of Algarve, Portugal

We would like to thank the reviewer for the encouraging evaluating of our systems biology approach and his suggestions for further improvement of the study and its presentation. In response to the comments, following changes to the manuscript were made:

1. In the Introduction, we pointed out the finding of a stress-sensitive association of HTT with the ER membrane, which suggests a potential direct role of HTT in ER stress. The relevant references are now included.
2. We clarified the meaning of the sentence. It states now: “As the UPR has also been associated with suppression of gene transcription and the enhanced degradation of numerous transcripts, ...”
3. We would like to thank reviewer for pointing out this data set. We analysed the correlation of expression changes with the length of polyglutamine tract. Notably, UPR genes were found to be strongly overrepresented among genes whose expression in the striatum significantly correlate with the length of polyglutamine tract. The correlation and the corresponding significance are also shown for the potential UPR-HD connectors in table 3 and Supplementary dataset 9. The results suggest length of the polyglutamine tract (which is reversely correlated with age of disease onset in human patients) plays a critical role in the activation of UPR. We aim to include these expression changes also in our newly developed web-portal UPRHD at http://uprhd.sysbiolab.eu.
